# Supplementary material for: Community-Acquired, Bacteraemic Acinetobacter Baumannii Pneumonia: A Retrospective Review of Cases in Tropical Queensland, Australia
Source: Trop Med Infect Dis. 2023 Aug 18;8(8):419. doi: 10.3390/tropicalmed8080419 (PMC10458713; doi:10.3390/tropicalmed8080419)
Supplement: Supplementary file 1 [file tropicalmed-08-00419-s001.zip › tropicalmed-2532403-supplementary.pdf]

Table S1: Antibiotic sensitivity profile of isolates

Isolate	GEN	MER	CIP	FEP	TAZ/TIM	CAZ	SXT	CRO
1	S	S	S	S	S	S	S	S
2	S	S	S	S	S	S	R	R
3	S	S	R	S	S	S	S	R
4	S	S	S	S	S	S	R	R
5	S	S	S	S	S	S	S	S
6	S	S	S	S	S	S	S	R
7	S	S	S	S	S	S	R	R
8	S	S	S	S	S	S	S	R
9	S	S	S	S	S	S	S	S
10	S	S	S	N/A	S	S	S	S
11	S	S	S	S	S	S	R	R
12	S	S	S	S	S	S	S	R
13	S	S	S	S	S	S	S	R
14	S	S	S	S	S	S	S	R
15	S	S	S	S	S	S	R	S
16	S	S	S	S	S	S	S	R
17	S	S	S	S	S	S	S	R
18	S	S	S	S	S	S	S	N/A
19	S	S	S	S	S	S	R	R
20	S	S	S	S	S	S	S	R
21	S	S	S	S	S	S	R	R
22	S	S	S	S	S	S	R	R
23	S	S	S	S	S	S	S	R
24	S	S	S	S	S	S	S	R
25	S	S	S	S	S	S	S	R
26	S	S	S	S	S	S	S	R
27	S	S	S	S	S	S	S	R
28	S	S	S	S	R	R	S	R

GEN = Gentamicin, MER = Meropenem, CIP = Ciprofloxacin, FEP = Cefepime, TAZ = Piperacillin-Tazobactam, TIM = Ticarcillin/Clavulanate, CAZ = Ceftazidime, SXT = Sulfamethoxazole-Trimethoprim, CRO = Ceftriaxone, N/A = Not Available.
